# Dynamic Filament Formation by a Divergent Bacterial Actin-Like ParM Protein

**DOI:** 10.1371/journal.pone.0156944

**Published:** 2016-06-16

**Authors:** Anthony J. Brzoska, Slade O. Jensen, Deborah A. Barton, Danielle S. Davies, Robyn L. Overall, Ronald A. Skurray, Neville Firth

**Affiliations:** 1 School of Life and Environmental Sciences, University of Sydney, Sydney, NSW, 2006, Australia; 2 School of Medicine, Ingham Institute for Applied Medical Research, University of Western Sydney, Sydney, NSW, 1871, Australia; University of Manchester, UNITED KINGDOM

## Abstract

*A*ctin-*l*ike *p*roteins (Alps) are a diverse family of proteins whose genes are abundant in the chromosomes and mobile genetic elements of many bacteria. The low-copy-number staphylococcal multiresistance plasmid pSK41 encodes ParM, an Alp involved in efficient plasmid partitioning. pSK41 ParM has previously been shown to form filaments *in vitro* that are structurally dissimilar to those formed by other bacterial Alps. The mechanistic implications of these differences are not known. In order to gain insights into the properties and behavior of the pSK41 ParM Alp *in vivo*, we reconstituted the *parMRC* system in the ectopic rod-shaped host, *E*. *coli*, which is larger and more genetically amenable than the native host, *Staphylococcus aureus*. Fluorescence microscopy showed a functional fusion protein, ParM-YFP, formed straight filaments *in vivo* when expressed in isolation. Strikingly, however, in the presence of ParR and *parC*, ParM-YFP adopted a dramatically different structure, instead forming axial curved filaments. Time-lapse imaging and selective photobleaching experiments revealed that, in the presence of all components of the *parMRC* system, ParM-YFP filaments were dynamic in nature. Finally, molecular dissection of the *parMRC* operon revealed that all components of the system are essential for the generation of dynamic filaments.

## Introduction

Recent advances in prokaryotic cell biology have challenged the long-held notion that bacterial cells exist merely as casings that contain diffusible chemicals and enzymes. Improved bacterial fluorescent imaging techniques, coupled with the abundance of publically available bacterial genome data, has enabled the spatio-temporal localization of novel proteins to be determined *in vivo*. Strikingly, bacteria contain an array of proteins which not only adopt specific localizations within cells, but form an integral part of a bacterial subcellular cytoskeleton [1]. For example, the bacterial cytoskeletal protein FtsZ, a distant homologue of eukaryotic tubulin, forms a distinct ring-shape at mid-cell (the ‘Z-ring’) that defines the prokaryotic divisional plane and recruits further proteins involved in bacterial cytokinesis, whereas the actin-like protein MreB that is found in rod-shaped cells forms a discontinuous helical structure involved in controlling the width of a bacterium during cellular growth [2]. While only a few examples of prokaryotic tubulin homologues have been found to date [1], genes encoding *a*ctin-*l*ike *p*roteins (Alps) are prevalent in the chromosomes and mobile genetic elements (such as plasmids) of many diverse bacterial species [3,4]. Phylogenetic analyses have revealed that chromosomally-encoded Alps, such as MreB, are closely related to each other, whereas Alps present on bacterial mobile genetic elements show vast inter-species sequence divergence [3]. Despite the genetic diversity exhibited by bacterial Alps, crystal structures from distantly related Alps have revealed that they share the basic ‘actin-fold’–the cleft present within all homologues of eukaryotic actin that is required for ATP/GTP binding and hydrolysis–and the ability of the Alp monomer to polymerize into filamentous ultrastructures [5].

**Fig 1:**
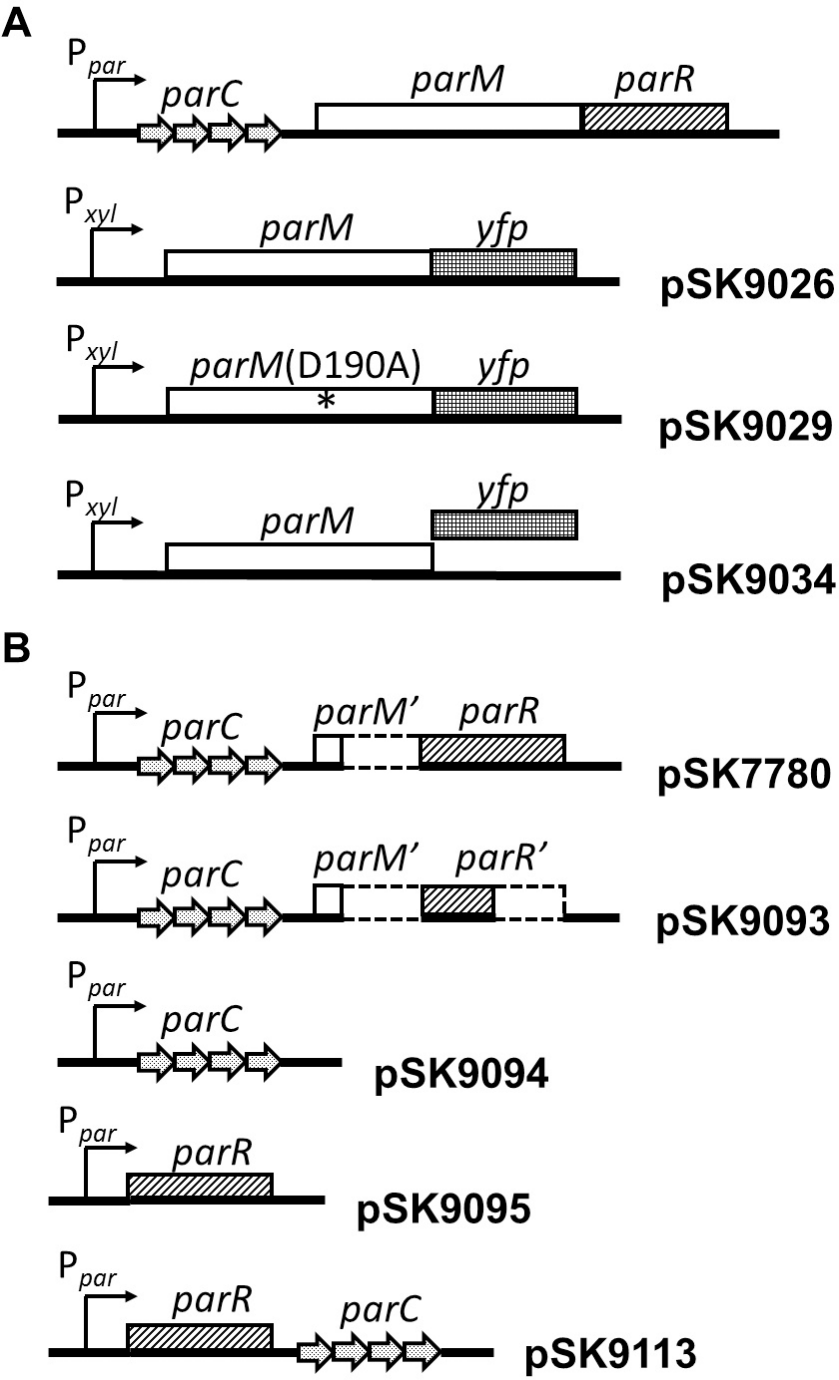
**Genetic structures of the pSK41 *parMRC* operon and *parM* plasmids (A), and *parRC* plasmid constructs generated in this study (B).** *parM* is shown as an open box, *parR* is shown as a cross-hatched box, and *yfp* is shown as gridded box. Repeats at *parC* are shown as stippled arrows. P_*par*_ and P_*xyl*_ are shown as arrows indicating the direction of transcription. Deleted sequences are denoted by dashed lines. The approximate location of the mutation in *parM-yfp* of pSK9029 that results in a D190A substitution is indicated by an asterisk. See text for details of plasmid construction. Diagrams are not to scale.

The 46 kb *Staphylococcus aureus* plasmid pSK41 harbors a genetic locus, *parMRC* (Fig 1A), that encodes an actin-like protein, ParM [6,7]. pSK41 is the prototype of a family of medically important conjugative staphylococcal multiresistance plasmids [8] that have most recently been implicated in the development of *vanA*-mediated vancomycin resistance in *S*. *aureus* [9]. We have previously shown that pSK41 *parMRC* significantly enhances the segregational stability of an unstable staphylococcal mini-plasmid, and site directed mutagenesis indicated that the NTPase motif of ParM is required for this stability phenotype [6]. The *parMRC* locus also encodes a DNA binding protein, ParR, which recognizes a series of 10 bp direct repeats, *parC*, located directly upstream from the *parM* and *parR* structural genes. Crystallographic data of ParR bound *parC* DNA shows that ParR binds as a dimer-of-dimers to the *parC* repeats, producing an extended macromolecular structure known as the ‘segrosome’ [6]. In the related *ParMRC* partitioning system of the *E*. *coli* multiresistance plasmid R1, ParM interacts with the segrosome to segregate replicated plasmids in a bidirectional fashion. *In vitro* data indicate that pSK41 ParM adopts a polymeric conformation which is very different to that of actin, MreB and R1 ParM [10]. Whereas pSK41 ParM forms a helical single stranded filament, both R1 ParM and actin adopt a two-start helical conformation, while MreB forms linear protofilaments [10]. Remarkably, database searching using pSK41 ParM crystal structure co-ordinates revealed that this protein is most structurally related to the chromosomally encoded Alp Ta0583 from the archaea *Thermoplasma acidophilum*, and not the R1 plasmid partitioning protein ParM, underscoring the structural diversity within microbial Alps. Biophysical analyses have suggested that pSK41 ParM filaments undergo a treadmilling-like mechanism of motion *in vitro* similar to that of F-actin [10]; contrastingly, R1 ParM exhibits a form of dynamic instability similar to that of eukaryotic tubulin [11]. *In vivo* studies in the native staphylococcal host, using a ParM C-terminal fusion to red fluorescent protein (ParM-RFP), also suggested that pSK41 ParM filaments are not dynamically unstable [12], in agreement with the *in vitro* observations [10]. Interestingly, the plasmid-partitioning Alp protein Alp7A, from the 55 kb *Bacillus subtilis* plasmid pLS20, exhibits both treadmilling and dynamic instability [3]. These observations highlight significant diversity in the dynamic properties exhibited by Alps.

Despite the elucidation of biochemical and biophysical characteristics of the divergent pSK41 ParM Alp [10], little is known about the nature of pSK41 ParM filaments *in vivo* [12]. Here, we have reconstituted the pSK41 *parMRC* system in the ectopic rod-shaped host *E*. *coli*, and have demonstrated that *parMRC* is functional in this organism, indicating that pSK41 *parMRC* is functional as a discrete unit. Using time-lapse fluorescent imaging and selective photobleaching microscopy, we also show that ParM polymers are dynamic in nature, and define the *par* system components required for this activity. This work enhances the understanding of prokaryotic Alps via the elucidation of the *in vivo* filament-forming properties of this highly divergent bacterial actin-like ParM protein.

## Materials and Methods

### Bacterial Strains, Plasmids, Growth Conditions, Media, and Reagents

Plasmid-containing *E*. *coli* DH5α cells (Bethesda Research Laboratories) were routinely cultured in Luria Broth (LB) containing 20 μg/ml chloramphenicol or 100 μg/ml ampicillin. Both antibiotics were used at the above concentrations for the selection of co-transformed strains. Liquid broth cultures were grown with agitation using a mechanical orbital shaker (New Brunswick Scientific), set to 220 rpm. Solid media culture was performed using standard non-vented petri plates (Sarstedt, Australia) containing LB supplemented with 1.5% agar and antibiotics where required. All bacterial cell culture was undertaken at 37°C. Reagents for cell culture were purchased from Oxoid (Australia), and chemical reagents were purchased from Sigma-Aldrich (Australia). Bacterial strains and plasmids used in this study are listed in S1 Table.

Plasmid pSK7780 was constructed using the multi-step process detailed below. A ~400 bp fragment encompassing the *parC* region was amplified from pSK41 plasmid DNA with the primers AB1 and orf346P2HindIII. Concurrent with this process, a second ~400 bp fragment, containing the full-length *parR* structural gene, was amplified from pSK41 DNA using the primers AB2BamHI and orf346P3HindIII. The purified AB1-orf346P2HindIII PCR product was digested with *Xba*I and *Hind*III, and the purified AB2BamHI-orf346P3HindIII product was digested with *Bam*HI and *Hind*III, before these fragments were co-ligated into pAM401, which had been digested with *Xba*I and *Bam*HI. The resultant plasmid, pSK7780, contains the pSK41 *parC* region and the *parR* open reading frame (ORF), and a deletion derivative of *parM* (*parM‘*), which expresses only the first 10 amino acids of the protein. Plasmids pSK9026, pSK9029, pSK9093, pSK9094 and pSK9113 were generated via standard cloning procedures using primers listed in S2 Table. pSK9095 was generated via the multi-step procedure described below. A PCR fragment encompassing P_*par*_ was amplified from pSK7780 using the primers AB1 and AB123. Concurrent with this process, a second PCR fragment harboring the *parR* ORF was amplified from pSK7780 using the primers AB122 and AB2BamHI. Primers AB122 and AB123 contain regions of complementarity, and these regions were used to join the two amplicons in a PCR reaction which included the primers AB1 and AB2BamHI. The fusion PCR product was digested with *Xba*I and *Bam*HI and was ligated into similarly prepared pAM401 plasmid DNA, giving rise to pSK9095.

### DNA manipulations

DNA manipulations were undertaken using standard protocols detailed in Sambrook *et al*. [13]. Plasmid DNA extraction from recombinant *E*. *coli* cultures was performed using the Bioline Isolate Plasmid Mini-Kit. Polymerase chain reaction (PCR) was done using iProof DNA High-Fidelity DNA Polymerase (Bio-Rad, Australia), using oligonucleotides synthesized by Geneworks, Australia. Primer sequences can be located in S2 Table. Restriction digestion was undertaken using reagents purchased from New England Biolabs (NEB), according to the manufacturer’s instructions. DNA fragments were purified when necessary using the Wizard SV® Gel and PCR Clean-Up system (Promega). Ligation was undertaken at 16°C for 16 hours using T_4_ DNA ligase purchased from NEB. Recombinant plasmid constructs were sequenced at the Australian Genome Research Facility’s (AGRF) Sydney node.

### Plasmid Segregational Stability Assays

Plasmid segregational stability assays were conducted according to a method modified from Schumacher *et al*. [6]. Briefly, plasmids to be assayed were grown overnight in LB supplemented with both ampicilln and chloramphenicol. The following morning, the stationary phase culture was diluted in 0.1% saline, and viable counts were performed using solid LB media containing ampicillin for the selection of ParM or ParM-Yellow Fluorescent Protein (YFP) fusion expressing plasmids. Using the saline diluted cultures, a 10^−4^ dilution was made into 10 ml fresh LB containing ampicillin, and was incubated overnight at 37°C with shaking. This process was repeated until approximately 50 generations of growth was achieved (5 days). 50 colonies from the viable count plates were patched onto ampicillin-chloramphenicol double selection media, and the proportion of plasmids remaining in the population was quantified. Stability assays were conducted using three biological replicates, and the standard errors of plasmid-retaining populations were determined using the statistical package available with Microsoft Excel 2007. Differences in plasmid segregational stabilities were evaluated by Fisher’s protected least-significant-difference test after repeated-measures ANOVA, using SPSS Statistics for Macintosh, Version 22.0 (IBM Corporation). A significant difference was defined as a *P* value of <0.05.

### Microscopy

*E*. *coli* strains to be assayed were grown with selection overnight. A 1:50 dilution of the saturated culture was made in fresh LB with selection, and the culture was grown to an OD_600_ nm of 0.6. 0.5 ml of the mid-logarithmic phase culture was harvested by centrifugation, and the pellet was washed once with 0.5 ml phosphate buffered saline (PBS). Cells were collected by centrifugation, and the pellet was resuspended in 50 μl PBS. 3 μl of the cell solution was applied to a 2% agarose pad set within a 65 μl Gene-Frame (Integrated Sciences). Cells were examined using a AxioImager Z1 fluorescence microscope (Carl Zeiss) equipped with a 100 X oil immersion objective lens with a numerical aperture of 1.4. Yellow Fluorescent Protein was excited using light passed through a bandpass 500/20 filter and emitted light was collected through a bandpass 535/30 filter. Images were captured using a Photometrics CoolSNAP HQ camera. Samples for selective photobleaching experiments were prepared as above and then imaged using a LSM 510 Meta confocal microscope (Carl Zeiss) with a 488 nm argon laser at 4% power. A 63 X oil immersion lens with a numerical aperture of 1.4 was used. Regions of interest were photobleached using five iterations of five laser lines (458, 477, 488, 514 and 561 nm), each at 100% power. To monitor recovery of fluorescence after photobleaching, the cells were imaged every 7.6 seconds for three minutes. To capture movies of filament dynamics over time, cells were imaged using a Nikon Eclipse Ti live cell imaging system and a 100 X oil immersion lens with a numerical aperture of 1.45. Excitation light from a LED source passed through a 509/22 filter and emitted light was collected through a 542/25 emission filter. Images were taken every 10 seconds over a 5 minute period in a time series using an Andor iXon Ultra 888 digital camera. To correct for sample drift in the x and y planes over time, each time series was aligned with the Linear Stack Alignment with SIFT plugin within FIJI (http://fiji.sc/Fiji). Movie files were exported at 10 frames per second.

## Results and Discussion

### Reconstituted *trans-acting* pSK41 *parMRC* is functional in the ectopic rod-shaped host, *E*. *coli*

Protein components derived from the *E*. *coli* plasmid R1 *parMRC* operon have been shown to retain mechanistic functionality in the presence of *parC* coated microspheres *in vitro* [14]. This suggests that *parMRC*-based partitioning systems are self-contained functional units [14]. In light of this observation, we sought to reconstitute the *parMRC* system from pSK41 in the heterologous rod-shaped bacterium *E*. *coli*, in order to study ParM filament formation and dynamics *in vivo*. We selected a rod-shaped organism for these studies, rather than the native coccoid host, since the orientation of assembled ParM filaments with respect to the plane of division is easily observable, thereby making it more amenable to functional analyses. Moreover, *E*. *coli* cells are larger than *S*. *aureus* cells, making them easier to visualize; are easier to genetically transform and manipulate; and have previously been used to study the filament dynamics of partitioning proteins [15]. A diagrammatic representation of the wild-type *parMRC* operon is shown in Fig 1A.

**Fig 2:**
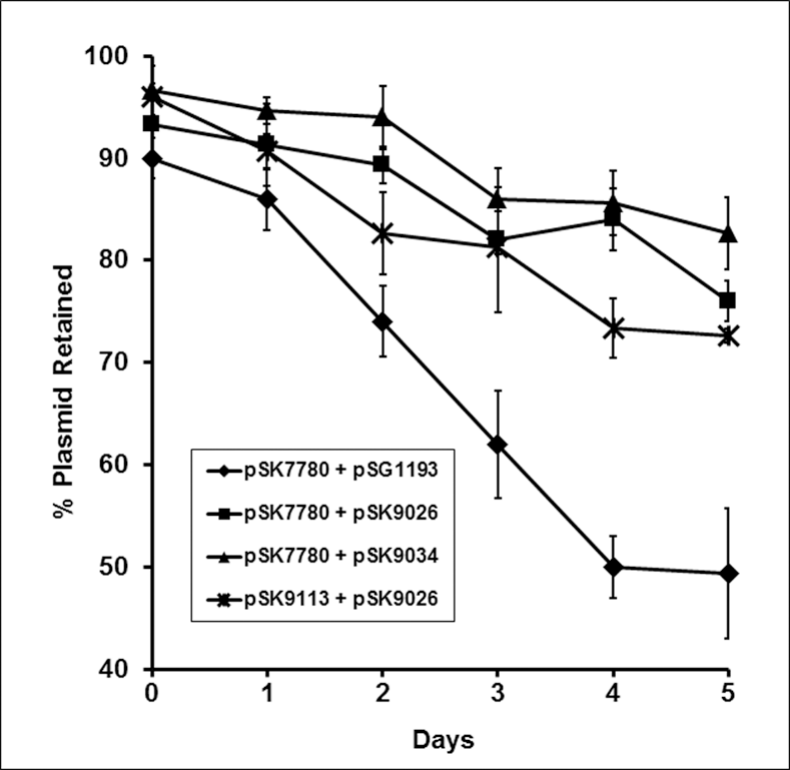
Segregational stability of *parC*-containing pSK7780 or pSK7780-derived test plasmids in the presence or absence of ParM or ParM-YFP in the *E*. *coli* DH5α cells. The retention of pSK7780, containing the *parC* centromere and *parR* transcribed from P_*par*_ was determined in the presence of pSK9026 (squares; expressing ParM-YFP), pSK9034 (triangles; expressing ParM which had been uncoupled from YFP), or pSG1193 (diamonds; expressing YFP only). The retention of pSK9113 (stars), containing *parR* transcribed from P_*par*_, but with *parC* cloned downstream from the *parR* ORF, was determined in the presence of pSK9026. Five days of serial subculture represents approximately 50 generations of growth. Each data point is the mean of three biological replicates. Standard error is shown.

To reconstitute pSK41 *parMRC* in *E*. *coli*, we constructed a *trans*-acting system that contains components of the *parMRC* system distributed across two plasmids. The two-plasmid system described here was necessitated because numerous attempts to clone the entire intact *parMRC* operon repeatedly resulted in plasmids that accumulate mutations when maintained in *E*. *coli*, usually in the *par* promoter or in the *parM* gene, indicating that, when expressed in its intact form, the operon is deleterious in *E*. *coli*. Plasmids constructed for this system harbor compatible *E*. *coli* replication systems, and contain complementary resistance markers. pSK9026 (Fig 1A) is a derivative of the *B*. *subtilis* integration plasmid pSG1193 that contains *parM* cloned as an in-frame fusion to the *yfp* open reading frame. On pSK9026, transcription of the *parM-yfp* fusion is controlled by the *B*. *subtilis* promoter P_*xyl*_, which is constitutive *in E*. *coli*. A site-directed mutagenesis derivative of pSK9026, in which *parM* was uncoupled from the *yfp* ORF, was also constructed (pSK9034; Fig 1A)). pSK7780 (Fig 1B) is a moderate copy-number plasmid that replicates using a p15A replicon [16], that contains *parC* and *parR*, but with a PCR-generated deletion in *parM*, so that only the first 10 amino acids of ParM is expressed. To assess the functionality of this reconstituted system, *E*. *coli* DH5α cells were co-transformed to ampicillin-chloramphenicol double resistance with pSK7780 and either pSK9026 (*parM-yfp*), pSK9034 (*parM*), or pSG9113 (*yfp*), and the retention of pSK7780 over approximately 50 generations of bacterial growth was determined using segregational stability assays. The assays were performed in the absence of selection for pSK7780, but included ampicillin to ensure the carriage of the other plasmids. These assays (Fig 2) revealed that the *parCR* plasmid pSK7780 was significantly more stably maintained in the presence of ParM (encoded by co-resident pSK9034; *P* = 0) or ParM-YFP (from pSK9026; *P* = 0) than it was in the absence of ParM (vector pSG1193 co-resident), thereby indicating that the reconstituted pSK41 *parMRC* system was at least partially functional in the ectopic *E*. *coli* host. Moreover, ParM-YFP (pSK9026) resulted in pSK7780 stability approximating that mediated by ParM (pSK9034; *P* = 0.2), suggesting that the YFP tag did not markedly impede ParM function. Although chromosomal segregation systems, which do not encode actin-like NTPases, have been shown to be able to stabilize plasmids in heterologous hosts [17,18], to our knowledge this is the first demonstration of an Alp-based plasmid partitioning system functioning in an ectopic host. Since Gram-positive and Gram-negative bacteria diverged from a common ancestor around 2 billion years ago [19,20], our *in vivo* data corroborate the *in vitro* observations of Garner *et al*. [14] (see above), which indicated that *parMRC*-like segregation systems are autonomous functional units.

### ParM-YFP forms filaments in heterologous host cells

**Fig 3:**
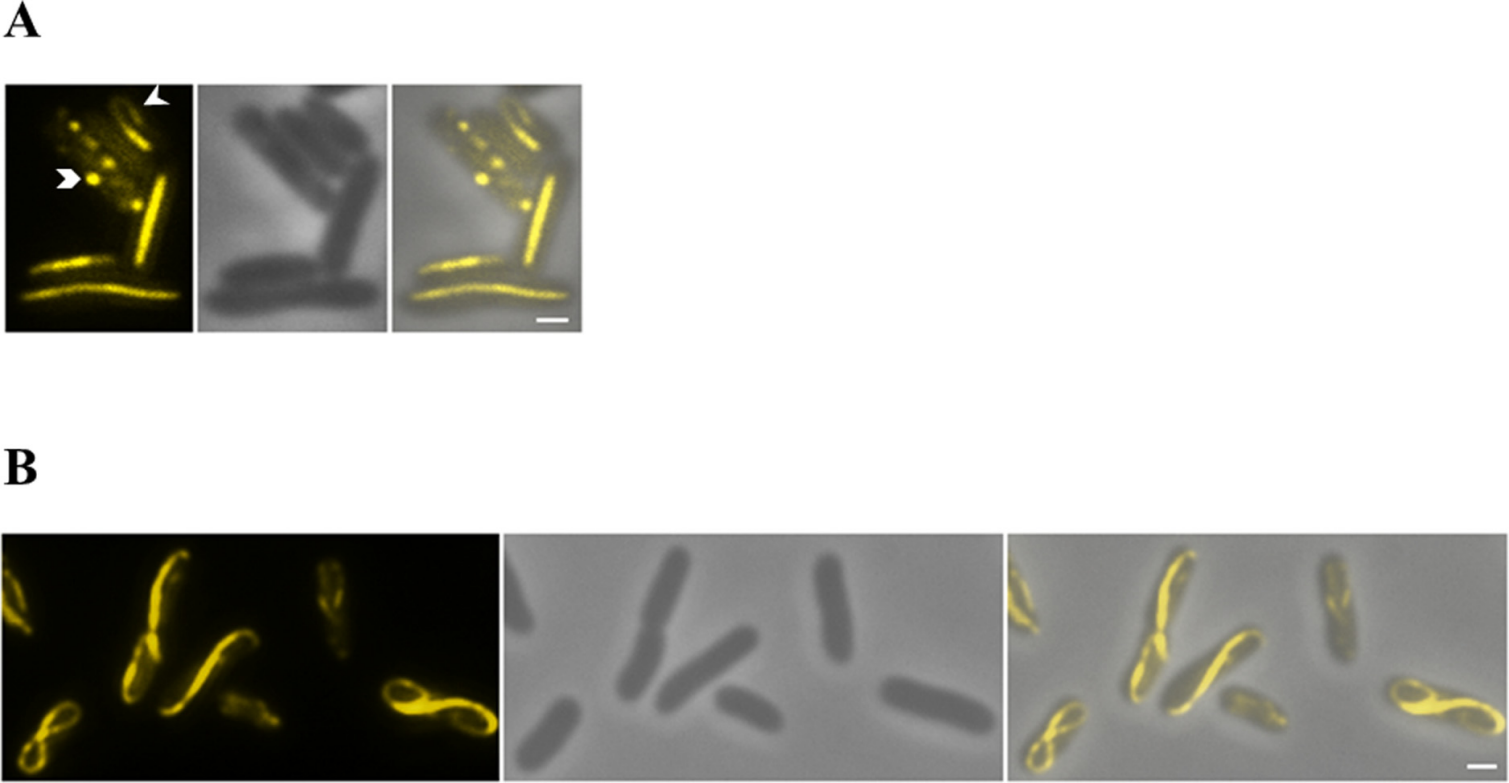
ParM-YFP filament formation in the presence or absence of *parC* and ParR. *E*. *coli* cells expressing ParM-YFP in isolation (A) or expressing ParM-YFP in the presence of *parC* and ParR (B) were visualized by fluorescence microscopy. ParM-YFP filaments exhibit a dramatic shift in morphology in the presence of *parC* and ParR. Hook shaped filaments are indicated with an arrow-head, and ParM-YFP foci are shown with a chevron. Left to right, both panels: fluorescence image, phase contrast image; merge of phase contrast and fluorescent images. Scale bars represent 1 μm.

In order to gain insights into the pSK41 *parRMC* partitioning mechanism *in vivo*, we conducted fluorescence microscopy using the *E*. *coli* strains expressing ParM-YFP described above. In isolation, ParM-YFP (expressed from pSK9026) produced straight pole-to-pole filaments in the majority of cells surveyed (Fig 3A); these are likely to represent filament bundles rather than individual ParM-YFP filaments. Fluorescent foci were present throughout the cytoplasm of occasional cells, possibly representing genesis points for ParM-YFP filaments. In some cells, longer ParM-YFP filaments appeared to curve around the perimeter of the cell, producing a hook-shaped polymer. Most cells contained a single ParM-YFP filament, however, a small number of cells appeared to contain two or three; none contained more. Thus, although longer, the ParM-YFP filaments observed in *E*. *coli* closely resembled the straight ParM-RFP filaments seen previously in the smaller coccoid natural host, *S*. *aureus* [12].

Since an intact NTPase motif in ParM is essential for the *in vivo* partitioning phenotype of the *parMRC* locus [6], we explored its requirement for ParM filament formation. To do this, we constructed a pSK9026 derivative that contains a *parM* ORF harboring a D190A mutation in the NTP binding domain [6,7], fused to *yfp* (pSK9029; Fig 1A; S1 Table). Interestingly, when pSK9029 containing cells were visualized by fluorescence microscopy, only general fluorescence was observed (data not shown). In contrast, equivalent mutations that abolished the ATPase activity of ParM from R1, and the plasmid segregation Alp, AlfA, from *B*. *subtilis* plasmid pLS32, did not abolish polymerization of those Alp proteins [7,21]. Although we cannot preclude the possibility that the D190A mutation may prevent proper protein folding, the lack of ParMD190A-YFP filaments might indicate that the D190A mutation in pSK41 ParM disrupts nucleotide binding rather than hydrolysis. However, further experiments are required to investigate the precise effect of the D190A mutation on the ATPase activity of pSK41 ParM. An ATPase deficient mutant of Alp7A was also unable to produce filaments at wild-type cellular concentrations, but was able to produce filaments when the protein was significantly overproduced [22]. In these cells, Alp7A forms large and amorphous polymers that interrupt chromosomal segregation and cell division. Although expression of pSK41 ParM-YFP and ParMD190A-YFP was unregulated in *E*. *coli*, cell morphology did not appear to be affected.

In order to assess pSK41 ParM-YFP polypeptide localization with other components of the *par* system present, *E*. *coli* cells harboring both pSK9026 (expressing ParM-YFP) and pSK7780 (Fig 1) were visualized by fluorescence microscopy. Strikingly, cells expressing ParM-YFP and containing the *parC* centromere-like site and ParR showed a dramatically different ParM-YFP filament morphology compared to cells expressing ParM-YFP alone. Most of the cells within this population contained thinner, curved fluorescent structures that extended along the axis of the *E*. *coli* cell, which sometimes appeared lemniscate (figure-eight shaped) in nature (Fig 3B); it is likely that these also represent filament bundles rather than individual filaments (see below). Importantly, since *E*. *coli* cells in which all of the components of the pSK41 partitioning system were reconstituted imparted a stable partitioning phenotype to test plasmids (Fig 2), this observation suggests that a shift in ParM filament morphology correlates with an active partitioning phenotype *in vivo*. Interestingly, the filament morphology of ParM-YFP in the presence of both *parC* and ParR differs to that of other characterized plasmid Alps; whereas pSK41 ParM-YFP often appeared to be continuous, ParM from R1, AlfA, and Alp7A exhibit a curved, but open-ended, filament shape [3,7,21]. However, as the curved ParM-YFP filaments observed here have been generated in an ectopic host, and since expression of the ParM-YFP fusion protein is unregulated, the observed lemniscate architecture of the polymer may not be representative of its typical conformation in its natural coccoid host at wild-type expression levels.

### Curved ParM-YFP filaments are dynamic

The plasmid partitioning Alps ParM from R1, AlfA, and Alp7A form dynamic polymers, and this characteristic is essential for the partitioning function exhibited by these systems [3,7,21]. Mechanistically, dynamic polymerization of plasmid segregation Alps can be achieved in a variety ways: ParM from plasmid R1 exhibits dynamic instability (analogous to that of eukaryotic tubulin); AlfA from pLS32 exhibits treadmilling (analogous to that of eukaryotic actin); and Alp7A from pLS20 exhibits *both* dynamic instability and treadmilling [3,11,23]. *In vitro* time-lapse total internal reflection fluorescence (TIRF) imaging using purified pSK41 ParM suggested that ParM polymers do not exhibit dynamic instability, and instead exhibit a treadmilling phenotype [24]. Consistent with this observation, live cell imaging of ParM-RFP filaments in the native *S*. *aureus* host indicated that ParM in isolation is not dynamically unstable *in vivo* [12]. In order to elucidate further mechanistic details of pSK41 ParM filaments in the bacterial cytosol, we conducted live-cell time lapse fluorescent microscopy imaging, using the ParM-YFP expressing strains described above. Images were captured for each ParM-YFP expressing strain every 10 seconds over a time course of five minutes, and images were processed and compiled into a motion picture. This analysis revealed that ParM-YFP filaments formed in the presence of *parC* and ParR (pSK7780) were dynamic (S1 Movie), whereas ParM-YFP filaments produced in isolation were static (S2 Movie). In all cells visualized, the curved ParM-YFP filaments appeared to undergo active remodeling during the capture period. Filaments did not adopt a preferred distribution within the cell, and polymers of varying lengths were observed forming and redistributing throughout the entire cell cytosol, indicating that ParM filaments are not compartmentalized or confined to particular cellular locales. Importantly, we did not observe any evidence of catastrophic disassembly *in vivo*, consistent with *in vitro* and *in vivo* evidence reported previously [10,12] that indicated that pSK41 ParM filaments do not exhibit dynamic instability [10,12].

**Fig 4:**
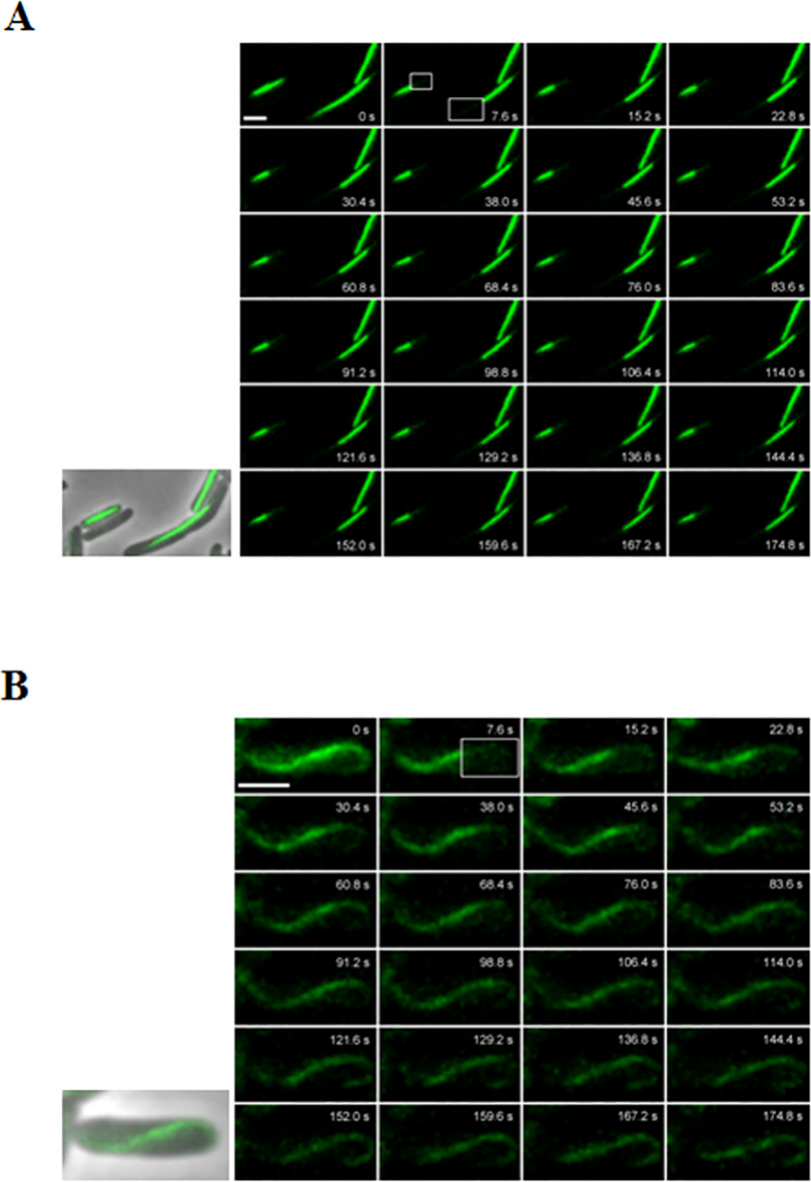
ParM-YFP filaments are dynamic in the presence of *parC* and ParR. *E*. *coli* cells expressing either ParM-YFP in isolation (A) or ParM-YFP in the presence of *parC* and ParR were grown to mid-logarithmic phase and selective photobleaching experiments were undertaken. Fluorescence recovery was monitored by imaging cells every 7.6 seconds over the course of three minutes. The first image in the series depicts a pre-bleached cell. Phase contrast/fluorescence overlay images of cells expressing ParM-YFP are shown to the left of the photobleaching montage. Boxes indicate regions of laser photobleaching. Time units (seconds; s) are shown. Scale bars represent 2 μm.

To gain further insights into the nature of pSK41 ParM-YFP polymer formation, we conducted selective photobleaching experiments, using the strains described above. Cells containing pSK9026 (ParM-YFP), or pSK9026 and pSK7780 (ParR and *parC*), were grown to mid-logarithmic phase prior to visualization via fluorescence microscopy. As expected, ParM-YFP filaments expressed in isolation showed no recovery of the photobleached areas over the course of the experiment (Fig 4A; S3 Movie), supporting evidence that ParM-YFP expressed in isolation are static (see above). In contrast, photobleached areas of ParM-YFP filaments formed in the presence of ParR and *parC* showed a rapid recovery, confirming that ParM-YFP monomers exhibit active turnover within dynamic ParM-YFP polymers (Fig 4B; S4 Movie). Fluorescent recovery to the right of the filament in Fig 4B (S4 Movie) appears to be at the expense of diminishing fluorescence to the left of the bleached region, consistent with dynamic filament turnover. Similar to AlfA, ParM-YFP bleached zones appeared to recover using the same track as observed prior to bleaching [25], which, as suggested by Polka *et al*., indicates that ParM protofilaments are likely to form a close lateral association with each other [25]. Likewise, cryoelectron tomography-based experiments have revealed that ParM from R1 forms bundles of three to five filaments that are actively involved in plasmid segregation, indicating that filament bundling is likely to be a common mechanism involved in Alp-mediated partitioning [26].

### ParM-YFP requires *parC* and ParR for dynamic ParM-YFP polymer turnover

Dynamic ParM-YFP filaments are generated in the presence of all components of the pSK41 *parMRC* system *in vivo* (see above). To delineate the requirements for ParM-YFP dynamic filament formation, a series of plasmids containing various components of the pSK41 *parMRC* system were constructed, in order to determine the contribution of each component individually, or in combination, to filament dynamics. Four constructs were made using the pAM401 parent vector, and separately co-transformed with pSK9026 (expressing ParM-YFP alone) into competent *E*. *coli* DH5α cells. The first plasmid generated, pSK9093 (Fig 1B), contains an amplicon encompassing P_*par*_ and *parC*, and a harbors a fragment of *parR* (*parR’*) engineered to express only the first 52 amino acids of the mature ParR polypeptide (ParRN). Our previous work has shown that ParRN is sufficient to be able to bind the pSK41 *parC* region [6]. pSK9093 was therefore able to be used to determine if the formation of the segrosome, in the absence of the C-terminus of ParR (which is thought to have a role in the recruitment of ParM proteins to the segrosome [6]), is sufficient to be able to generate dynamic ParM-YFP polymers *in vivo*. pSK9094 (Fig 1B) contains a fragment encompassing only *parC* and the P_*par*_ promoter, and pSK9095 (Fig 1B) contains the entire *parR* ORF, transcribed by P_*par*_, but lacks *parC*. Since *parR* transcription is autoregulated via ParR binding to *parC* [6], any phenotype observed with pSK9095 might be attributable to unregulated *parR* expression rather than an absence of *parC* itself (e.g., for assembly of the ParR-*parC* segrosome). pSK9113 (Fig 1B), containing *parC* downstream of *parR*, was therefore constructed to allow these possibilities to be unambiguously differentiated. Thus, any differences in ParM-YFP behavior observed between cells harboring pSK9095 or pSK9113 can be attributed to the presence/absence of *parC*, and not unregulated *parR* expression.

Plasmids generated were separately co-transformed with pSK9026 into *E*. *coli* DH5α cells, and time-lapse microscopy (as detailed above) was undertaken. The results of these studies showed that ParM-YFP filaments in the presence of pSK9093 (*parCR’*), pSK9094 (*parC*), or pSK9095 (*parR*), were largely static (S5, S6 and S7 Movies). However, in strains harboring pSK9113, which therefore contain the entire reconstituted *parMRC* system, ParM-YFP active polymer turnover was evident (S8 Movie). Segregational stability assays, which determined the retention of pSK9113 in the presence of pSK9026 (*parM-yfp*) over the course of approximately 50 generations of growth, showed that pSK9113 is more stable than the parent plasmid pSK7780 co-resident with the *yfp* vector pSG1193 (*P* = 0.001), indicating that the reorganized *parRC* system on pSK9113 retains functionality (Fig 2). Thus, these results indicate that 1) all components of the pSK41 *parMRC* region are required for dynamic ParM-YFP filament turnover; 2) the C-terminus of ParR is essential for function of the segregation system; and 3) the interaction of *parC*, ParR, and ParM, to generate the pSK41 segregation complex, activates ParM-YFP dynamic filament formation. These findings are consistent with the assembly mechanism proposed by Gayathri *et al*. [27], who showed that a 17 amino acid region from the C-terminus of R1 ParR interacts directly with the polymerization interface of the ParM polymer. Based on structural observations, these authors hypothesize that ParR monomers are required to be released from the ParM-ParR complex for ParM polymerization to occur, and that the ParR-*parC* complex, composed of ten ParR dimers, forms a scaffold that facilitates ParM polymerization via a ‘stair-stepping’ mechanism, analogous to that of eukaryotic formin. It is unknown, at this stage, whether such a ‘stair-stepping’ mechanism for filament polymerization is universally conserved amongst all bacterial Alps.

It should be noted that the *parMRC* system reconstituted here in *E*. *coli* represents a very different context to the native system on pSK41 in *S*. *aureus*. In particular, in addition to autoregulation by ParR [6], transcription from the *par* promoter is controlled by a global regulator of plasmid transcription encoded by pSK41, ArtA [28]. Moreover, pSK41 is a large conjugative plasmid with a tightly-controlled narrow-host-range replication system [29], and at least two other plasmid maintenance determinants; viz., the *res* multimer resolution system [30] and a *fst*-like toxin-antitoxin system [31]. Remarkably, isolated from all these extrinsic factors in an unrelated host, the reconstituted minimal pSK41 *par* system was still able to perform its basic biological function, increasing the segregational stability of a *parC*-containing plasmid. However, when considering the observations described here it should be remembered that they are, by necessity, of an artificial system involving multiple plasmids, heterologous promoters, a YFP fusion to ParM, and an unrelated host; but they are nonetheless observations of a functional partitioning system. As noted above, the two-plasmid system described here was employed because attempts to clone the entire *parMRC* operon in *E*. *coli* resulted in plasmids that accumulate mutations, indicating that, when expressed in its intact form, the operon is deleterious in *E*. *coli*. The basis for this toxicity is not clear but the absence of ArtA control might be a contributing factor. In this regard, it should be noted that P_*par*_ doesn’t drive transcription of *parM* in the two-plasmid system used in these studies. Additionally, a non wild-type ParM protein (ParM-YFP) was used to track the distribution of ParM proteins within the *E*. *coli* cytosol. Caution must be exercised when interpreting the distribution of ParM-YFP filament bundles, since YFP is known to dimerize in *in vivo* [32,33]. The dimerization properties of fluorescent protein tags have recently been implicated in the aggregation of fused polypeptides *in vivo* [33,34,35,36]. Nonetheless, it would seem unlikely that YFP dimerization could be responsible for the *parCR*-dependent morphological shift in ParM-YFP filament bundles observed, particularly in view of the plasmid stabilizing activity of the reconstituted system in the absence of any native ParM.

In summary, we have shown that a YFP-tagged derivative of the staphylococcal pSK41 *parMRC* system is functional in the heterologous *E*. *coli* host, and that the formation of dynamic ParM-YFP filaments correlates with partition function *in vivo*. All components of the *parMRC* system were required for the generation of dynamic ParM-YFP filaments. Moreover, the C-terminus of ParR, which facilitates the recruitment of ParM to the segrosome complex, was shown to be required for the conversion of static ParM filaments to a dynamic form proficient for active segregation. This study adds further important information to the suite of growing data elucidating the *in vivo* properties of the diverse array of recently described bacterial Alps.

## Supporting Information

S1 Movie ParM-YFP is dynamic in the presence of *parC* and ParR. *E*. *coli* cells expressing ParM-YFP in the presence of *parC* and ParR (expressed from pSK7780) were grown to mid-logarithmic phase and fluorescence microscopy was undertaken. Images were captured every 10 seconds over a time course of 5 minutes. Images were compiled into a motion picture using FIJI.
(AVI)

S2 Movie ParM-YFP polymers are static in the absence of ParR and *parC*. *E*. *coli* cells expressing ParM-YFP in the absence of ParR and *parC* were grown to mid-logarithmic phase and fluorescence microscopy was undertaken. Images were captured every 10 seconds over a time course of 5 minutes. Images were compiled into a motion picture using FIJI.
(AVI)

S3 Movie Photobleached regions of ParM-YFP polymers do not recover in the absence of ParR and *parC*. *E*. *coli* cells expressing ParM-YFP in the absence of ParR and *parC* were grown to mid-logarithmic phase and fluorescence microscopy was undertaken. Regions of interest were photobleached using five iterations of five laser lines (458, 477, 488, 514 and 561 nm), each at 100% power. Recovery of fluorescence of photobleached cells was monitored by imaging every 7.6 seconds for three minutes. Captured images were processed using ImageJ v1.48.
(MOV)

S4 Movie Photobleached regions of ParM-YFP polymers show rapid recovery in the presence of ParR and *parC*. *E*. *coli* cells expressing ParM-YFP in the presence of ParR and *parC* were grown to mid-logarithmic phase fluorescence microscopy was undertaken. Regions of interest were photobleached using five iterations of five laser lines (458, 477, 488, 514 and 561 nm), each at 100% power. Recovery of fluorescence of photobleached cells was monitored by imaging every 7.6 seconds for three minutes. Captured images were processed using ImageJ v1.48.
(MOV)

S5 Movie ParM-YFP polymers are static in the presence of a truncated ParR protein. *E*. *coli* cells expressing ParM-YFP in the presence of *parC* and a truncated ParR protein (ParRN; expressed from pSK9093) were grown to mid-logarithmic phase and fluorescence microscopy was undertaken. Images were captured every 10 seconds over a time course of 5 minutes. Images were compiled into a motion picture using FIJI.
(AVI)

S6 Movie ParM-YFP polymers are static in the absence of ParR. *E*. *coli* cells expressing ParM-YFP in the presence of *parC* (on pSK9094), but in the absence of ParR, were grown to mid-logarithmic phase and fluorescence microscopy was undertaken. Images were captured every 10 seconds over a time course of 5 minutes. Images were compiled into a motion picture using FIJI.
(AVI)

S7 Movie ParM-YFP polymers are static in the absence of *parC*. *E*. *coli* cells expressing ParM-YFP in the presence of ParR (expressed from pSK9095), but in the absence of *parC*, were grown to mid-logarithmic phase and fluorescence microscopy was undertaken. Images were captured every 10 seconds over a time course of 5 minutes. Images were compiled into a motion picture using FIJI.
(AVI)

S8 Movie The entire *parMRC* system is required to produce active ParM-YFP polymers. The *parMRC* system was reconstituted using plasmid pSK9113 so that the expression of ParR is under the control P_*par*_ and *parC* is present downstream from the *parR* ORF. pSK9026, expressing ParM-YFP, was co-transformed with pSK9113 and resulting strains were grown to mid-logarithmic phase and fluorescence microscopy was undertaken. Images were captured every 10 seconds over a time course of 5 minutes. Images were compiled into a motion picture using FIJI.
(AVI)

S1 Table Strains and plasmids used in this study. (DOC)

S2 Table Oligonucleotides used in this study. (DOC)
