# Supplementary material for: Dynamic Filament Formation by a Divergent Bacterial Actin-Like ParM Protein
Source: PLoS One. 2016 Jun 16;11(6):e0156944. doi: 10.1371/journal.pone.0156944 (PMC4911067; doi:10.1371/journal.pone.0156944)
Supplement: S1 Table — (DOC) [file pone.0156944.s009.doc]

Table S1. Strains and plasmids used in this study.

| **Strain/Plasmid** | **Genotype/Description*^a^*** | **Source/Reference** |
| --- | --- | --- |
| ***E. coli* Strains** |  |  |
| DH5α | F^–^ *endA hsdR17 supE44 thi-1 *λ^-^ *recA1 gyrA96 relA1* φ80 *ΔlacZ*ΔM15 | Bethesda Research Laboratories |
| **Plasmids** |  |  |
| pSK41 | Nm^R^, Ble^R^, Eb^R^, Qac^R^, Gm^R^, Tm^R^, Km^R^, Tra^+^. *S. aureus* conjugative multiresistance plasmid. | Wood *et al*., 1977[37] |
| pAM401 | Cm^R^, Tc^R^, p15A *ori*, pIP501 *ori*. *E. faecalis-E. coli* shuttle plasmid. | Wirth *et al.*, 1986[38] |
| pSG1193 | Ap^R^, Sp^R^, ColEI high copy *ori*, P*_xyl_* MCS-*yfp.* | Feucht & Lewis, 2001[39] |
| pSK7780 | Cm^R^. *par* region with a PCR generated in-frame deletion of *parM* cloned as an *Xba*I-*Bam*HI fragment into pAM401. | This work |
| pSK9017 | Ap^R^, Nm^R^, pSK41 minireplicon containing the *parMRC* region but with a D190A substitution within the *parM* ORF | Schumacher *et al*., 2007 |
| pSK9026 | Ap^R^, Sp^R^, pSG1193 with *parM* cloned as a *Kpn*I-*Eco*RI fragment to create an in-frame C-terminal fusion with YFP. | This work |
| pSK9029 | Ap^R^, Sp^R^, pSG1193 with *parMD190A* cloned as a *Kpn*I-*Eco*RI fragment (amplified from pSK9017) to create an in-frame C-terminal fusion with YFP. | This work |
| pSK9034 | Ap^R^, Sp^R^, pSK9026 with *parM* uncoupled from *yfp* using site-directed mutagenesis | This work |
| pSK9093 | Cm^R^. pAM401 containing *parC* and a truncated *parR* gene, which expresses the first 52 amino acids of ParR (ParRN), amplified from pSK7780 and cloned as an *Xba*I-*Bam*HI fragment. | This work |
| pSK9094 | Cm^R^. pAM401 containing *parC* cloned as an *Xba*I-*Bam*HI fragment. | This work |
| pSK9095 | Cm^R^. pAM401 harboring a PCR generated fusion fragment which contains *parR* downstream from P*_par_*, cloned into the *Xba*I and *Bam*HI sites of the vector. | This work |
| pSK9113 | Cm^R^. pSK9095 with *parC* cloned downstream from *parR*, into the *Bam*HI and *Sph*I sites of the vector. | This work |
|  |  |  |

*^a^*Nm^R^: neomycin resistance; Ble^R^: bleomycin resistance; Eb^R^: ethidium bromide resistance; Qac^R^: quaternary ammonium compounds resistance; Gm^R^: gentamicin resistance; Tm^R^: tobramycin resistance; Km^R^: kanamycin resistance; Ap^R^: ampicillin resistance; Cm^R^: chloramphenicol resistance; Tc^R^: tetracycline resistance; Sp^R^: spectinomycin resistance; Tra^+^: conjugative ability; *ori*: origin of replication
