# Supplementary material for: Dynamic Filament Formation by a Divergent Bacterial Actin-Like ParM Protein
Source: PLoS One. 2016 Jun 16;11(6):e0156944. doi: 10.1371/journal.pone.0156944 (PMC4911067; doi:10.1371/journal.pone.0156944)
Supplement: S2 Table — (DOC) [file pone.0156944.s010.doc]

Table S2. Oligonucleotides used in this study.

| **Name** | **Sequence*^a^*** | **Source/Reference** |
| --- | --- | --- |
| AB1 | ccg**tctaga**tccatggaaagtatgtttg | Schumacher *et al*., 2007 |
| Orf346-P2 | acc**aagctt**cataataaaacctccac | This work |
| Orf346-P3 | gcg**aagctt**aaagtagagcaagaaag | This work |
| AB2-*Bam*HI | atat**ggatcc**aaatggaattggatgaagg | This work |
| AB20 | g**gaattc**tttactttcttgctctactttg | This work |
| AB23 | cc**ggtacc**atgagtaacgtatatgtaatg | This work |
| AB120 | atat**ggatcc**ttatattggagggttttcccc | This work |
| AB121 | atat**ggatcc**aattatttactttcttgctctac | This work |
| AB122 | gctggttgttatttttttacccacacttgtggtattttattaattgtac | This work |
| AB123 | gtacaattaataaaataccacaagtgtgggtaaaaaaataacaaccagc | This work |
| AB131 | atat**ggatcc**tccatggaaagtatgtttg | This work |
| AB132 | atat**gcatgc**aattatttactttcttgctctac | This work |

***^a^***Restriction sites are highlighted in bold.
